# Fighting antimicrobial resistance in a resource-limited setting: an integrated approach to infection prevention and antimicrobial stewardship is the ‘best buy’

**DOI:** 10.1016/j.jhin.2025.02.023

**Published:** 2025-07

**Authors:** B.D. Fofanah, I.F. Kamara, R.Z. Kamara, R. Musoke, S.M. Tengbe, L. Kabego, S. Lakoh

**Affiliations:** a Emergency Preparedness and Response Cluster, World Health Organization Country Office, Freetown, Sierra Leone; b Disease Prevention and Control, Ministry of Health, Freetown, Sierra Leone; c Department of Internal Medicine, College of Medicine and Allied Health Sciences, University of Sierra Leone, Freetown, Sierra Leone; d Emergency Preparedness and Response, World Health Organization, Regional Office for Africa, Cité du Djoué, P.O. Box 06, Brazzaville, Congo

The compounding problem of healthcare-associated infection (HAI) and antimicrobial resistance (AMR) remains a global health concern as they prolong hospital stays, increase healthcare costs, and cause long-term disability and avoidable deaths [1]. Of the 1.27 global bacterial AMR deaths in 2019, 63.5% were due to HAI [2]. A recent report estimated that there are 136 million healthcare-associated AMR infections annually [3]. Sub-Saharan African countries bear the highest burden attributable to bacterial AMR [4]. Sierra Leone reported 2200 deaths attributed to bacterial AMR and 9700 associated AMR deaths, making AMR-related deaths even higher than cardiovascular diseases, and maternal and neonatal disorders [5].

Antimicrobial stewardship (AMS) to optimize the use of antimicrobial drugs and minimize AMR should be combined with effective infection prevention and control (IPC) as the cornerstone of actions to control AMR and the spread of multidrug-resistant pathogens. This interwoven relationship between IPC and AMR cannot be overstated. Effective IPC measures can abate the need for antimicrobials, while AMS guarantees that when antimicrobials are required they are used optimally to prevent selection pressure. A recent *Lancet* series estimated that improving IPC programmes in low- and middle-income country (LMIC) healthcare settings could prevent at least 337,000 (7.8%) AMR-associated deaths annually, surpassing that of Water, Sanitation, and Hygiene (WASH) and vaccination interventions [6]. Therefore, it is important to establish and implement complementary IPC and AMS programmes, especially in LMICs where the burden of AMR is high, and many hospitalized patients are given antibiotics. Where feasible, IPC and AMS programmes are recommended to address the gaps in human resources and the integration of services. In our previous study where we found a high prevalence (73.7%) of antibiotics use and a lack of AMS programmes, we recommended leveraging the already established IPC committees in these hospitals to create combined IPC and AMS committees [7]. This approach is feasible in our setting and can be explored using the experience of the national referral hospital in establishing and operationalizing a combined AMS and IPC programme [8]. An integrated IPC and AMS committee can develop joint action plans and guidelines, train healthcare workers, and conduct antibiotic use surveys and clinical evaluations.

In this article, we reviewed the joint reports (through a short online survey) of hospital pharmacists and IPC focal points in October 2024 from six secondary and six tertiary hospitals across the five regions of Sierra Leone. These hospitals were conveniently selected because they had both hospital pharmacists and IPC focal points and were part of the first national antibiotic use point-prevalence survey and training in Sierra Leone.

The average bed capacity is 192, and the hand hygiene compliance rate was 34%. This survey included selected indicators of the WHO AMS toolkit and IPC core components checklists that we considered relevant to covering the basic standards of infrastructure, policy, and practice for IPC and AMS [9,10].

*Infrastructure:* Although all 12 hospitals indicated the presence of IPC programmes, only three (25%) had established a formal AMS committee. All the hospitals with established AMS committees are tertiary facilities. Six (50%) of the hospitals reported they had access to a functional microbiological laboratory, whether within the hospital or externally. Of these facilities, five (83%) were tertiary hospitals. Among the three hospitals with an AMS committee, only one has a combined IPC/AMS committee.

*Policy and practice:* A considerable number of hospitals have implemented policies, guidelines, and frameworks aimed at monitoring and surveillance of IPC indicators. However, most hospitals lack the essential elements of hospital AMS such as AMS education and training (11, 92%), lack of antibiotic formulary list (100%), lack of prescription/treatment guidelines or policies (11, 92%), and lack of surveillance of AMR and antibiotic utilization (10, 83%). It is noteworthy that the Connaught Hospital, which had established a joint IPC and AMS committee, was the only facility reported to have substantially accomplished numerous facets of infrastructure, policy, and practice on AMS and IPC (Table I).

**Table I:** Performance of IPC and AMS indicators reported by hospital IPC focal points and pharmacist across 12 hospitals in October 2024

Indicators	Tertiary care						Secondary care					
	MH34	PCMH	KEGH	CONH	ODCH	BOGH	MGH	KMCH	BNGH	KOGH	KAGH	MAGH
Infrastructure												
Formal AMS programme with a multidisciplinary committee/team present	Yes	No	No	Yes	Yes	No	No	No	No	No	No	No
Appointed and trained AMS champion or focal point	Yes	No	No	Yes	Yes	No	No	No	No	No	No	No
An IPC programme with trained IPC focal point	Yes	Yes	Yes	Yes	Yes	Yes	Yes	Yes	Yes	Yes	Yes	Yes
A functioning IPC committee/team	Yes	Yes	Yes	Yes	Yes	Yes	Yes	Yes	Yes	Yes	Yes	Yes
Functional microbiological laboratory at the hospital/access outside the hospital	Yes	Yes	Yes	Yes	Yes	No	No	No	No	Yes	No	No
Policy and practice												
Updated antibiotic formulary based on the Essential Drug List and WHO AWaRe	No	No	No	No	No	No	No	No	No	No	No	No
Antibiotic prescription/treatment guidelines/policy^a^	No	No	No	Yes	No	No	No	No	No	No	No	No
Updated national IPC guidelines are available	Yes	Yes	Yes	Yes	Yes	Yes	Yes	Yes	Yes	Yes	Yes	Yes
Hand hygiene compliance monitored in the last 3 months (%)^b^	Yes (22)	Yes (44)	Yes (45)	Yes (47)	Yes (40)	Yes (32)	Yes (36)	Yes (46)	Yes (24)	Yes (44)	Yes (16)	Yes (13)
Surveillance of HAI is undertaken	No	No	Yes^c^	Yes^c^	No	Yes^c^	Yes^c^	Yes^c^	Yes^c^	No	Yes^c^	Yes^c^
Antibiotic use/consumption monitored and reported^d^	No	No	No	Yes	Yes	No	No	No	No	No	No	No
Hospital collects data on AMR and antibiotic susceptibility as part of national AMR surveillance	No	No	No	Yes	Yes	No	No	No	No	No	No	No
Frontline health workers received training on AMS	No	No	No	Yes	No	No	No	No	No	No	No	No
HAI prevention bundles developed/implemented	No	No	No	No	No	No	No	No	No	No	No	No

IPC, infection prevention and control; AMS, antimicrobial stewardship; AWaRe, Access, Watch and Reserve; AMR, antimicrobial resistance; HAI, healthcare-associated infection; MH34, 34 Military Hospital; PCMH, Princess Christian Maternity Hospital; KEGH, Kenema Hospital; CONH, Connaught Hospital; ODCH, Ola During Children Hospital; BOGH, Bo Hospital; MGH, Moyamba Hospital; KMCH, Kingharman Maternal & Child Hospital; BNGH, Bonth Hospital; KOGH, Koidu Hospital; MAGH, Magburaka Hospital.

a Guideline/Policy: based on written prescription on documentation of indication for all antibiotic prescriptions, and preauthorization system, and AwaRe.

b Average hand hygiene compliance in last three months expressed in percentage (%).

c Surgical site infection surveillance.

d Antibiotic use or consumption (in grams by defined daily dose) is monitored, audited, or reviewed, and communicated directly with prescription.

This underscores the notion that the enhancement of IPC structures facilitates the seamless initiation and deployment of AMS programmes/committees within hospital settings. We therefore recommend that hospitals without AMS programmes leverage the existing IPC committees to fast-track the creation of AMS programmes. Studies have shown that AMS interventions to reduce the incidence of multidrug-resistant infections were more effective when implemented with IPC measures [11]. Further, when member countries of the OECD (Organisation for Economic Co-operation and Development) invest in IPC interventions such as improved hand hygiene and enhanced healthcare environmental hygiene, along with AMS, they generate healthcare savings that are higher than the implementation cost of the intervention [12].

In conclusion, implementing integrated IPC and AMS programmes in health facilities is feasible and likely the best approach to addressing AMR, especially in LMICs with scarce resources. Recognizing the need for further research in this area, it should be implemented taking into consideration the contextual situation and operational reality.

## Ethics approval

Not required.

## Funding sources

None.

## Conflict of interest statement

None declared.
